# Exercise medicine for cancer cachexia: targeted exercise to counteract mechanisms and treatment side effects

**DOI:** 10.1007/s00432-022-03927-0

**Published:** 2022-01-27

**Authors:** Georgios Mavropalias, Marc Sim, Dennis R. Taaffe, Daniel A. Galvão, Nigel Spry, William J. Kraemer, Keijo Häkkinen, Robert U. Newton

**Affiliations:** 1 Exercise Medicine Research Institute, Edith Cowan University, 270 Joondalup Drive, Joondalup, WA 6027 Australia; 2 School of Medical and Health Sciences, Edith Cowan University, Joondalup, Australia; 3 Institute for Nutrition Research, Edith Cowan University, Joondalup, Australia; 4 Medical School, University of Western Australia, Perth, Australia; 5 Department of Human Sciences, Ohio State University, Columbus, USA; 6 Neuromuscular Research Center, Faculty of Sport and Health Sciences, University of Jyväskylä, Jyvaskyla, Finland

**Keywords:** Cancer cachexia, Inflammation, Tumor, Exercise, Muscle wasting, Muscle atrophy

## Abstract

**Purpose:**

Cancer-induced muscle wasting (i.e., cancer cachexia, CC) is a common and devastating syndrome that results in the death of more than 1 in 5 patients. Although primarily a result of elevated inflammation, there are multiple mechanisms that complement and amplify one another. Research on the use of exercise to manage CC is still limited, while exercise for CC management has been recently discouraged. Moreover, there is a lack of understanding that exercise is not a single medicine, but mode, type, dosage, and timing (exercise prescription) have distinct health outcomes. The purpose of this review was to examine the effects of these modes and subtypes to identify the most optimal form and dosage of exercise therapy specific to each underlying mechanism of CC.

**Methods:**

The relevant literatures from MEDLINE and Scopus databases were examined.

**Results:**

Exercise can counteract the most prominent mechanisms and signs of CC including muscle wasting, increased protein turnover, systemic inflammation, reduced appetite and anorexia, increased energy expenditure and fat wasting, insulin resistance, metabolic dysregulation, gut dysbiosis, hypogonadism, impaired oxidative capacity, mitochondrial dysfunction, and cancer treatments side-effects. There are different modes of exercise, and each mode has different sub-types that induce vastly diverse changes when performed over multiple sessions. Choosing suboptimal exercise modes, types, or dosages can be counterproductive and could further contribute to the mechanisms of CC without impacting muscle growth.

**Conclusion:**

Available evidence shows that patients with CC can safely undertake higher-intensity resistance exercise programs, and benefit from increases in body mass and muscle mass.

## Introduction

Among the most detrimental side effects of cancer and treatment is cachexia, a multifactorial metabolic and immune system imbalance (Tisdale 2005). Cancer cachexia (CC) is the ongoing skeletal muscle loss (with or without fat mass loss) during cancer manifestation and treatment, which cannot be reversed by conventional nutritional support, leading to progressive functional impairment and death (Fearon et al. 2011). Half of all cancer patients develop cachexia, and this estimate increases to 80% in hospitalized or advanced-stage patients (Tisdale 2003). Cachexia is observed in 80% of gastric, pancreatic, and esophageal, ~ 70% of head-and-neck, ~ 60% of lung, colorectal, lymphoma, and prostate, and 54% of malignant pleural mesothelioma cancer patients (Laviano and Meguid 1996). Moreover, CC is the immediate cause of death of at least 22% of all cancer patients (Argilés et al. 2014). Notably, aside from cancer, cachexia is observed in the late stages of almost every major chronic illness (Farkas et al. 2013), such as HIV/AIDS (prevalence 35%), chronic heart failure and chronic obstructive pulmonary disease (20%), chronic kidney disease (40%), and rheumatoid arthritis (10%) (von Haehling and Anker 2010). Despite the prevalence and severity, cachexia remains under-researched, while treatment options are limited, due to treatment inadequacy and inconsistency (Roeland et al. 2020).

**Fig. 1:**
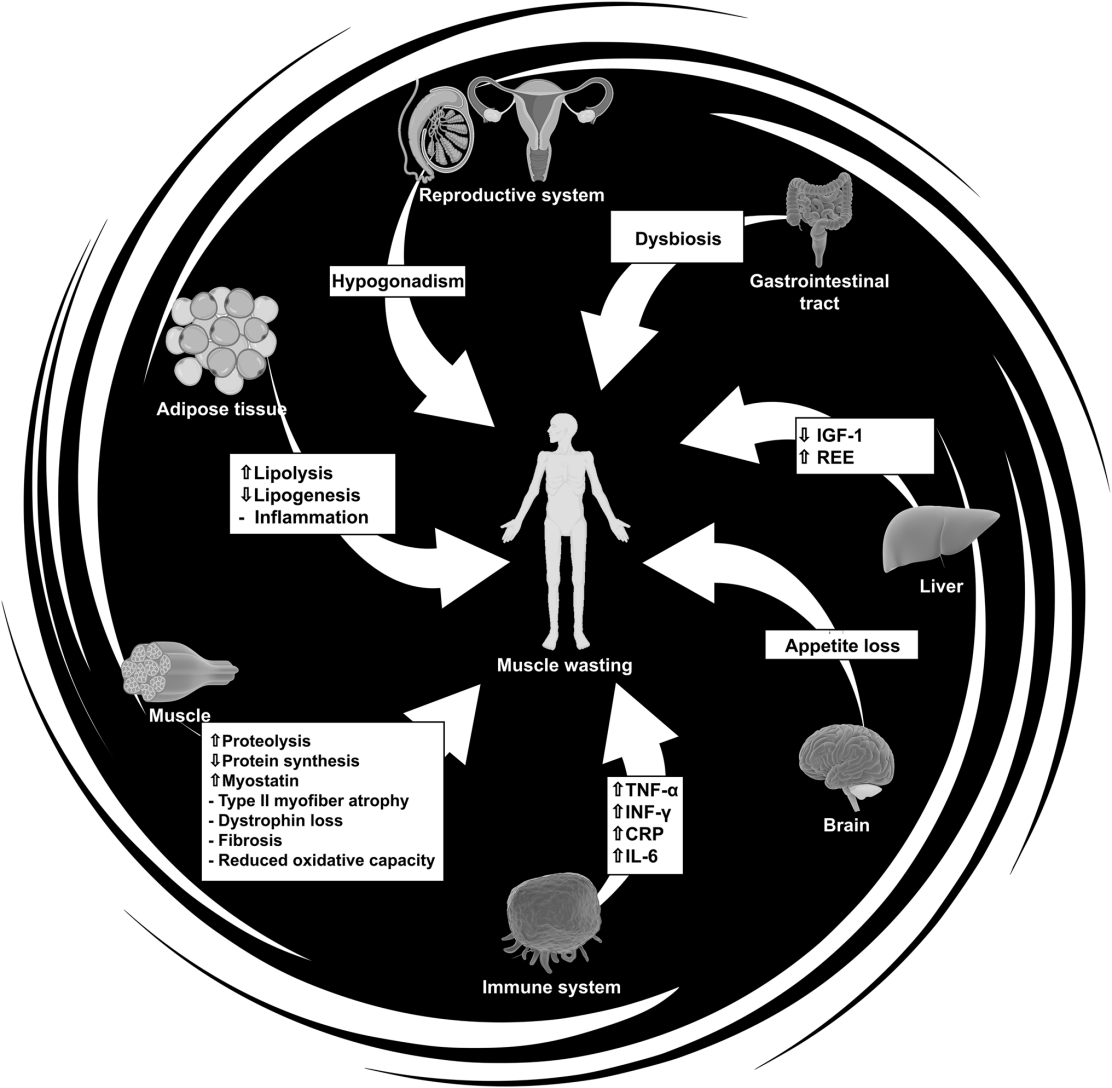
Cachexia mechanisms. *APR* acute phase response, *CRP* C-reactive protein, *ECM* extracellular matrix, *IGF-1* insulin growth factor 1, *IL* interleukin (1–11), *INF-γ* interferon gamma, *LMF* lipid-mobilizing factor, *PIF* proteolysis-inducing factor, *REE* resting energy expenditure, *TGF-β* transforming growth factor beta, *TNF-α* tumor necrosis factor alpha

CC progression is often described as a continuum, advancing from pre-cachexia to cachexia, and finally to refractory cachexia, where the expected survival is less than 3 months (Fearon et al. 2011). Even though its pathologic mechanisms are complex (see Fig. 1), it is often mistakenly regarded as a uniform condition, with little understanding that the underlying causes can be heterogeneous. Causes of CC can be malnutrition/anorexia (Fredrix et al. 1990), elevated inflammation (Tisdale 2005; Argilés et al. 2014), or even treatments such as chemotherapy (Brierley et al. 2019). Due to the complexity and varying proportion of underlying causes, a one-size-fits-all approach cannot be assumed, and different treatment strategies must be employed to counteract the patient’s mechanism profile.

While pharmacological interventions to reduce inflammation, stimulate appetite, or reduce muscle wasting already exist (Saeteaw et al. 2020), these should ideally be accompanied with adjunct non-pharmacological treatments, such as exercise, to amplify treatment effectiveness. Awareness that exercise as a medicine can be effective and reliable in cancer supportive care is well-established (Schmitz et al. 2019); however, there is a lack of understanding that exercise is not a single medicine, but mode, type, dosage, and timing (exercise prescription) have distinct effects on the components of health and fitness. For example, resistance exercise training (RET) consists of high-tension muscle contractions against a heavy external load and when performed regularly and in sufficient volume leads to increased muscle mass and strength (Grgic et al. 2019). On the other hand, endurance exercise consists of long durations of low-tension muscle contractions which result in increased respiration, cardiac output, and blood flow, leading to increased oxidative capacity, improved cardiovascular function, and fatigue resistance (Egan and Zierath 2013). Therefore, the different exercise modes will be reviewed to explore potential applications of targeted exercise medicine for CC management.

Apart from different exercise modes, different types of each mode can also elicit distinct and clinically-relevant outcomes. For example, during RET, different contractions are performed, such as concentric, eccentric, and isometric. Eccentric (lengthening) contractions are performed when the force generated by the muscle is less than the external load, causing the muscle to lengthen while resisting the external load. In contrast, concentric (shortening) contractions in which the force is greater than the load, allows the muscle to shorten (Vogt and Hoppeler 2014). During exercise composed of eccentric contractions (eccentric RET), the same muscle work can be produced with only ~ 15% of the metabolic demand of equivalent concentric RET, which enables the performance of more contractions for the same effort (Lastayo et al. 1999). Similarly in endurance training, due to cancer-related fatigue and reduced physical capacity, non-continuous training (interval) with short bursts of work and longer rests might be more tolerable than a single continuous effort. Moreover, higher-intensity interval endurance training (HIIET) is as effective at increasing muscle oxidative capacity as lower-intensity continuous endurance training (LICET) (Gibala and McGee 2008).

Recently, the American Society of Clinical Oncology published their guideline on the management of CC and concluded that exercise after the onset of CC is ineffective (Roeland et al. 2020), and therefore not recommended. These recommendations are particularly surprising, given that they were based on no trials (Roeland et al. 2020). Even in animal CC models, RET increases body (Donatto et al. 2013) and muscle mass (Hardee et al. 2016). Moreover, RET trials in cancer patients with particularly aggressive CC forms (e.g., pancreatic cancer) already exist (Table 1). Notably, RET did not only preserve muscle mass in patients with pancreatic and lung CC (Naito et al. 2019), but even increased body (Wiskemann et al. 2019) and muscle mass (Kamel et al. 2020) in patients with pancreatic CC, and increased muscle mass in head and neck cancer patients undergoing radiotherapy with large (> 8.5%) body mass loss (Lønbro et al. 2013a). Therefore, CC patients may experience clinically-significant muscle mass and strength gains following supervised RET (Lønbro et al. 2013a; Naito et al. 2019; Wiskemann et al. 2019; Kamel et al. 2020). However, more trials are needed to examine the most effective RET type, and the specific exercise parameters (i.e., intensity, volume, time under tension) to simultaneously increase muscle mass and reduce inflammation.

**Table 1:** Exercise training trials involving humans with cancer cachexia

Study	Population	Cachexia criteria	Intervention	Outcomes
Bland et al. (2021)	162 cancer patients	6-month BM loss at baseline was 10.4%; 7 (4%) patients had pre-cachexia, 83 (51%) had cachexia, and 29 (18%) had refractory cachexia	Multidisciplinary clinical service for cancer cachexia; same as Vaughan et al. (2020)	Stabilized BM between 6-week visits to the clinic, improved physical function, pain, nausea, appetite, anorexia-cachexia symptoms, physical, emotional and functional wellbeing
Capozzi et al. (2016)	60 head and neck cancer patients undergoing radiotherapy		12-week lifestyle intervention and progressive RET	The program failed to reduce loss of lean BM (− 5 kg) but improved quality of life, depression, and nutritional scores
Del Fabbro et al. (2011)	151 patients with cancer cachexia	History of BM loss ≥ 5% (median was 9%)	Dietary counseling by a dietician and standard exercise recommendations in an exercise clinic	Increased appetite and BM for those who returned for a second visit
Denehy et al. (2020)	45 patients with inoperable lung cancer	41% had CC	6-week biweekly home-based LICET	Better physical strength in adherent than non-adherent group, without differences in quality of life and disease symptoms
Grote et al. (2018)	12 head and neck cancer patients undergoing radiotherapy	BM loss (7.1%)	At least 13 sessions of RET (3 sets; 8–12 RM)	Increases in muscle strength but difference between groups in lean BM was not significant (intervention: + 1%, usual-care: − 3%)
Kamel et al. (2020)	Patients with pancreatic CC	BM loss > 5% over the past 6 months	12-week (2 week^−1^) whole-body RET (50–80% of the participant’s 1-RM; 3 sets; 8–12 repetitions)	Improvements in mobility, muscle mass, and strength, of both upper- and lower-limbs over a non-exercising group
Lønbro et al. (2013b)	21 head and neck cancer patients undergoing radiotherapy	Large BM loss > 8.5% in 2 months	12-week (30 sessions) whole-body RET with or without creatine and protein supplementation	5% increase in lean BM for the supplementation and 2.8% for the exercise-only group. Both increased strength
Lønbro et al. (2013a)	36 head and neck cancer patients undergoing radiotherapy	Large BM loss > 8.5% in 2 months	12-week (30 sessions) whole-body RET (2–3 sets of 8–15 RM)	4.3% increase in lean BM and increased muscle strength
Naito et al. (2019)	Advanced pancreatic and lung cancer scheduled for chemotherapy	BM loss of > 5% during the preceding 6 months or > 2% in patients with a BM index < 20 kg/m^2^; CC in 40% of patients	8 weeks of nutritional counseling, supplementation (branched-chain amino acids, coenzyme Q10, and L-carnitine) home-based bodyweight RET 3 sets of 10 repetitions	Body and skeletal muscle mass, and muscle function were maintained
Niels et al. (2018)	Case-study of stage IV pancreatic cancer patient undergoing chemotherapy	Typically expected 30% BM loss in patient	12-week biweekly RET (8–12 repetitions and 2 sets with 70–80% of rep-max), and LICET (70–80% of maximum of watt) 16 min, 2 sets	Maintained BM, increased strength
Rogers et al. (2013)	15 head and neck cancer patients undergoing radiotherapy	BM loss	12-week RET (exercise bands); 6-week supervised; 6-week unsupervised	Usual-care group lost 5.5 kg of lean BM, while intervention group lost only 0.4
Kaasa et al. (2015)**, **Solheim et al. (2017)**, **Balstad et al. (2020)	Pancreatic or lung cancer commencing chemotherapy	BM index < 30 kg/m^2^; and < 20% BM loss in the previous 6 months	6 weeks of (a) anti-inflammatory medication, (b) EPA supplementation, (c) nutritional counseling, (d) biweekly home-based LICET and RET	Even though only control group lost BM, both lost muscle mass
Storck et al. (2020)	52 advanced cancer patients		12-week leucine-rich supplementation, nutrition, and exercise program	Increases in lean BM did not reach significance vs usual-care. Increases in handgrip strength, trend for improvement in nutritional status, dietary intake, fatigue, quality of life and clinical course
Vaughan et al. (2020)	99 cancer patients	6% of patients were pre-cachectic (BM loss < 5%), 64% were cachectic (BM loss ≥ 5% or BMI < 20 with BM loss > 2%, systemic inflammation), and 30% had refractory cachexia (survival < 90 days, BM loss ≥ 5% or BMI < 20 with weight loss > 2%)	6-week home-based RET (5 exercises; ~ 4 week^−1^), high energy and protein diets, supplementation of fish oil, zinc, and multi-vitamins	49% displayed positive outcomes with > 2-kg BM gain between two consecutive appointments, 54% increased mid-upper arm muscle circumference, and > 50% improved functional strength between two consecutive appointments
Wiskemann et al. (2019)	65 patients with pancreatic cancer	Half of the patients had BM loss (≥ 10% in last 6 months)	6-months (2 week^−1^) whole-body RET either at home or performed under supervision in an exercise clinic (50–80% of 1-RM, 3 sets; 8–12 repetitions)	Higher adherence when home-based (78.4%) versus clinic-based (64.1%), but only the clinic-based group significantly increased upper- and lower-body strength, and BM (3.1%) over a non-exercising group

*BM* body mass, *CC* cancer cachexia, *LICET* low-intensity continuous endurance training, *RET* resistance exercise training, *RM* repetition maximum

The purpose of this review is to examine the most prominent CC mechanisms, and provide a rationale for research recommendations on specific exercise modes (and types) that could be used as a targeted non-pharmacological therapy integrated with the patient’s clinical treatment plan to not only reduce the side effects of cancer treatments but also improve their effectiveness, and reduce disease severity by reversing the multiple physiological mechanisms driving CC.

### Muscle mass wasting and increased protein turnover

The most devastating symptom of CC is muscle wasting, which can result from a variety of mechanisms (Fig. 1). Muscle mass is sustained by an intricate balance of protein breakdown and synthesis, known as protein turnover. Muscle proteins are in a constant state of turnover to maintain protein homeostasis, but CC disrupts this process, as there is simultaneously excessive protein breakdown and suppressed protein synthesis (White et al. 2013). Although increased body mass is desirable during CC, it is more important for the gained mass to be composed of muscle rather than fat. Specifically, skeletal muscle is an important resource for cancer patients, not only for metabolic, hormonal, and physical capacity reasons, but also because low muscle mass significantly predicts chemotherapy-induced toxicity and survival (Pin et al. 2018).

It is well established from a variety of studies that RET stimulates myofibrillar protein synthesis, whereas endurance training stimulates mitochondrial synthesis (Grgic et al. 2019). Consequently, endurance exercise training does not promote the same degree of skeletal muscle hypertrophy as RET (Grgic et al. 2019). However, recent reviews on exercise during CC have surprisingly recommended endurance over RET for preventing muscle wasting during CC (Aquila et al. 2020). In fact, when performing LICET concurrently with RET, it can result in smaller muscle growth compared to RET alone due to physiological interference (Wilson et al. 2012). For example, we have reported that prostate cancer patients who underwent androgen-deprivation therapy and RET, had greater increases in appendicular muscle mass versus those that included additional 20–30 min of LICET (Newton et al. 2019). Nevertheless, studies with preclinical models showed that endurance exercise might prevent muscle loss (Jee et al. 2016), however, only the mice undergoing higher-intensity activity (90% of maximum heart rate, every second day exercise for 45 min) preserved their muscle weight, while moderate intensities (70% of maximum heart rate) did not elicit the same effects (Jee et al. 2016). Moreover, lack of adequate intensity could have led to null findings in a study where patients with lung and pancreatic CC undergoing radiotherapy underwent 6-weeks of home-based exercise and supplementation (Solheim et al. 2017). The program consisted of twice-weekly LICET (30 min) and thrice-weekly RET, however, the exercises performed were of very light loads, such as body-weight pushups against the wall, and failed to significantly reduce muscle wasting (Solheim et al. 2017). Overall, current evidence suggests that heavier muscle loading is preferable and likely to be essential for hypertrophy.

During CC, metabolic and signaling pathways that increase protein synthesis are suppressed while pathways that decrease protein synthesis are activated, with this phenomenon considered the primary mechanism for muscle wasting (Tisdale 2009). Specifically, type II myofiber atrophy is particularly prevalent during CC, occurring to a greater degree than type I myofiber atrophy (Mendell and Engel 1971). Moreover, it is well-established that mTOR complex 1 (mTORC1) plays a central role in mechanical load-induced muscle growth by activating downstream substrates such as p70S6k, which is an integral pathway for muscle protein synthesis (Goodman et al. 2011). This pathway is regulated by signaling molecules such as insulin-like growth factor 1 (IGF-1), which is progressively decreased in tissue and blood during CC (White et al. 2013; Martins et al. 2018). However, it appears that this problem is multi-faceted, as exogenous IGF-1 treatment does not attenuate CC-induced muscle wasting (Costelli et al. 2006). Additionally, pathways that suppress protein synthesis, such as those involving AMPK, FoxO, STAT3, and myostatin are up-regulated during CC (White et al. 2013; Hardee et al. 2016, 2020). Previous investigations showed that eccentric RET stimulates pathways commonly affected during CC that cause muscle hypertrophy (IGF-1, mTORC1, p70S6k) and suppressed pathways that cause atrophy (FoxO, AMPK, STAT3, MuRF-1, myostatin) (Hardee et al. 2016, 2020; Tatebayashi et al. 2018; Martins et al. 2018), and those effects are often greater compared to equivalent concentric RET. Additionally, greater increases in type II myofiber size are observed from eccentric compared to concentric or even conventional RET (both concentric + eccentric), at least in healthy humans (Hather et al. 1991; Hortobágyi et al. 1996, 2000; Friedmann et al. 2004; Friedmann-Bette et al. 2010; English et al. 2014; Horwath et al. 2019). CC reduces muscle protein synthesis, partially through elevated IL-6 (Tisdale 2005; Argilés et al. 2014). Nevertheless, 14 sessions of maximal eccentric RET effectively increased protein synthesis (p70S6K and rpS6), reversed inhibitors of protein synthesis (MuRF-1), and reduced the CC-induced atrophy in mouse gastrocnemius (Tatebayashi et al. 2018). This was also verified by another group, as eight sessions of maximal eccentric RET improved oxidative metabolism, reduced muscle wasting, and increased basal muscle protein synthesis and mTORC1, and surprisingly, these improvements were positively correlated with plasma IL-6 levels (Hardee et al. 2020). These findings have significant implications for clinical practice due to the potential of repeated RET (particularly eccentric) in ‘exploiting’ elevated inflammation to proportionately increase muscle growth (see also “Systemic inflammation”).

Apart from signaling factor changes, CC can induce long-term muscle composition changes. For example, in mice with CC, muscle non-contractile tissue (fibrosis) was ~ 2.1-fold greater compared to healthy controls, but 2 weeks (4 week^−1^) of eccentric RET reduced fibrosis by 20% (Hardee et al. 2016). Another muscle-wasting mechanism during CC is dystrophin loss without an inherent genetic issue, leading to myofiber integrity impairments, muscle protein breakdown and wasting (Acharyya et al. 2005). In contrast, increased muscle integrin concentration can compensate for the lack of dystrophin in dystrophic animals by maintaining muscle mobility and structure and increasing muscle mass (Burkin et al. 2005). Integrin concentrations in humans increase after long-term eccentric RET (Mavropalias et al. 2021b), but there is a lack of information regarding the effects of concentric RET. We have recently reported that 20–30 min of eccentric RET per week elicited large increases (in some cases > 30%) in muscle cross-sectional area in healthy men after only 8 weeks (Mavropalias et al. 2021b). Given that integrins sense mechanical tension and stimulate protein synthesis, increased integrin concentration following eccentric RET could enhance anabolic signaling, thereby further amplifying muscle growth (Burkin et al. 2005).

Thus, eccentric RET appears to be specific and potent for counteracting the multiple causes of wasting at the myofiber level and could be very beneficial if incorporated as an additional brief component after a conventional RET program.

### Systemic inflammation

Persistently elevated circulating levels of interleukins, C-reactive protein (CRP), tumor-necrosis factor alpha (TNF-α), and interferon-γ are hallmark indicators and primary drivers of CC (Tisdale 2005; Argilés et al. 2009, 2014). These tumor-driven cytokines cause multiple health issues, such as anorexia, and increased metabolic rate, lipolysis, and proteolysis, among others (Tisdale 2005; Argilés et al. 2009, 2014). Therefore, controlling systemic inflammation is critical for CC prevention and management.

Exercise is generally thought to induce a pro-inflammatory state for a few hours following a session and thus may seem contra-indicated in the presence of already exacerbated inflammation. However, pro-inflammatory cytokines, such as TNF-α and interleukin-1, do not markedly increase after exercise, suggesting that the exercise-induced inflammatory profile differs from that induced by disease. In fact, muscle-derived IL-6 following exercise may inhibit the effects of pro-inflammatory cytokines such as TNF-α (Pedersen et al. 2001). Therefore, acute (a few hours post-exercise) exercise-induced increases in cytokine levels do not exacerbate already high-inflammation states but may instead exert an inflammation-controlling effect.

IL-6 is a critical cytokine for muscle metabolism, as it mediates muscle growth demonstrated by both in-vivo and in-vitro studies (Serrano et al. 2008). However, when chronically elevated, circulating IL-6 negatively correlates with myofiber cross-sectional area and protein synthesis (Hardee et al. 2020). Exercise training can lower elevated IL-6, as 12 weeks (3 week^−1^) of either descending or ascending stair-walking in women decreased resting IL-6 levels (− 24%) (Chow et al. 2020). In pre-clinical CC models, muscle growth induced by 2 weeks (4 week^−1^) of eccentric RET positively correlated with circulating IL-6 (Hardee et al. 2020), such that higher serum IL-6 was associated with greater muscle protein synthesis. This suggests that in mice with CC, repeated eccentric RET might mediate muscle protein synthesis through inflammation (greater inflammation induces greater muscle protein synthesis), a finding which may hold potential for CC patients. It is currently unknown if this effect is induced by concentric or conventional RET, however, based on its ability to control IL-6 in healthy women (Chow et al. 2020), pathways that mediate protein synthesis through elevated IL-6 might also be activated during concentric RET.

CRP is the most practical, cost-effective, and scientifically robust CC biomarker, with important roles in prognosis, and utility to predict quality of life in CC patients (Fearon et al. 2011; Laird et al. 2016). The results of one meta-analysis were that RET reduces CRP levels, but only when the programs included more than eight exercises, were performed at least 3 week^−1^, and for longer than 12 weeks (Sardeli et al. 2018). However, 1 year of biweekly RET was also effective in reducing CRP levels by ~ 10% in women (Olson et al. 2007). Regarding type, RET either with eccentric or concentric movements reduced CRP levels (25%) in sedentary humans, however, the authors reported that eccentric RET was significantly more efficient (~ 2.5 times) than concentric after adjusting for energy expenditure (Zeppetzauer et al. 2013). Specifically for cancer, a meta-analysis showed that a combination of RET and endurance training can reduce CRP levels (Khosravi et al. 2019). Regarding endurance exercise, patients with different cancer types had a 6% reduction in CRP levels after 12 weeks (3 week^−1^) of HIIET, but this was significantly different to the LICET group who exhibited a 19% increase (Toohey et al. 2016). These findings show a clear beneficial effect of RET of all types in reducing CRP levels in both healthy and cancer patients, however, there appears to be a preference for HIIET over LICET in cancer patients, although the information at this stage is limited.

Chronically elevated TNF-α, both circulating and in muscle, is a common symptom during CC, and has been also shown to be inversely associated with muscle protein synthesis (Greiwe et al. 2001; Argilés et al. 2009, 2014). However, 3 months (3 week^−1^) of lower-body RET was effective in reducing TNF-α levels in the muscle of frail elderly individuals (Greiwe et al. 2001). In another study, 12 weeks (3 week^−1^) of descending stair walking (eccentric exercise for knee extensors) was more effective (− 40%) than ascending stair walking training (− 24%) at decreasing TNF-α levels in women (Chow et al. 2020). While research on humans is limited, non-RET endurance training can precipitate decreased TNF-α levels, however, intensity matters, as 8 weeks (5 week^−1^) of HIIET was more effective than LICET in reducing TNF-α in mouse renal tissue (Leite et al. 2021).

Elevated circulating interferon-γ is another commonly-observed disease sign during CC (Argilés et al. 2009). Interferon-γ is produced by activated T and natural killer cells, and animal studies have shown that increased interferon-γ production rapidly develops CC, and that CC can be reversed by blocking interferon-γ (Argilés et al. 2009). In elderly women, 12 weeks (3 week^−1^) of whole-body light-load (elastic bands) RET reduced interferon-γ levels (12%) (Roh et al. 2020). Moreover, patients with prostate cancer decreased interferon-γ levels after only 8 weeks (3 week^−1^) of RET (Papadopoulos et al. 2021). Although further information regarding responses to long-term exercise training is currently limited, these results are promising and suggest that even light-load RET might effectively reduce interferon-γ levels.

Therefore, RET appears to be effective in reducing elevated TNF-α, CRP, and interferon-γ, while simultaneously ‘exploiting’ elevated IL-6, typically observed during CC, to increase muscle protein synthesis. When combined with HIIET, the inflammation-controlling effect may be potentiated.

### Reduced appetite and anorexia

Reduced appetite and anorexia contribute greatly to CC, especially during head-and-neck, gastrointestinal, and colorectal cancer (Fredrix et al. 1990). In fact, the disease profile of CC can appear similar to starvation, however, muscle wasting often precedes decreased energy intake, and can occur even without anorexia, in both humans and animals (Tisdale 2001).

Both acute sessions and 12 weeks (3 week^−1^) of exercise involving eccentric RET decreased preference and implicit wanting for sweet foods, but increased preference for fatty foods (Thivel et al. 2020). This might be particularly beneficial during CC, as patients are urged to consume an energy-dense diet composed of high amounts of fat (Arends et al. 2021). Equivalent concentric RET increased hunger and desire to eat more than eccentric, even though both concentric and eccentric RET equally increased total energy consumption (Thivel et al. 2020). Speculatively, due to the lower energy expenditure of eccentric RET (Lastayo et al. 1999), the increased energy consumption might result in a higher net energy balance compared to concentric. These findings suggest that eccentric might be preferable to concentric RET in simultaneously increasing preference for energy-dense foods and energy consumption, however, it is unknown if this applies during CC.

Regarding endurance training, post-exercise appetite or ad libitum energy consumption were unchanged after a single session of either LICET or HIIET (Poon et al. 2018). However, after 16 weeks (5 week^−1^), both LICET and HIIET, when combined with RET, increased fasting hunger, desire to eat, and total energy consumption in adolescents (Miguet et al. 2020). In another study, 12 weeks (3 week^−1^) of either HIIET or LICET increased fasting and postprandial feelings of hunger with no differences between type, however energy consumption was unchanged (Martins et al. 2017). One possible reason for this discrepancy is that the second study did not include RET (Martins et al. 2017). Even though more research is needed, especially during CC, these results suggest that a combination of RET and endurance training might increase hunger and total energy consumption.

### Increased energy expenditure and fat wasting

Despite reduced physical activity, energy expenditure increases during CC (Fredrix et al. 1990). This hyper-metabolic state is thought to occur from a combination of elevated inflammation and resting lipolysis (Fredrix et al. 1990). When combined with appetite reductions, it inevitably leads to fat and muscle wasting. Apart from being amplified by the increased energy expenditure, fat wasting is primarily induced by increased lipolysis, as there is an increased turnover of free fatty acids and glycerol, caused by elevated TNF-α (Mathur and Pedersen 2008; Tisdale 2009). Exercise increases energy expenditure; thus, CC management should include exercise modes that promote muscle growth with the lowest energy expended.

Endurance training has almost double the energy expenditure compared to RET, when matched for relative intensity (Bloomer 2005). Moreover, due to its capacity to induce white adipose tissue browning, mitochondrial biogenesis, fat loss, and thermogenesis, which are existing problems during CC (Grgic et al. 2019), LICET is less optimal during CC. Surprisingly, recent reviews recommended endurance training over RET to reduce muscle wasting during CC (Aquila et al. 2020). However, due to the above-mentioned contra-indications, LICET may be suboptimal against muscle wasting during CC. Nevertheless, in some cases, even higher-intensity endurance training modalities of shorter durations may be beneficial. For example, in mice with CC, 10 weeks of higher-intensity continuous endurance exercise (5 week^−1^, 30 min/day, 85% *V*O_2max_) increased lifespan, reduced tumor mass, and prevented reductions in total body fat (Bacurau et al. 2007). However, there is a lack of information on endurance training interspaced with rest on energy expenditure in CC patients. Interestingly, in healthy runners replacing LICET with HIIET did not impair aerobic capacity or muscle oxidative capacity, but decreased energy expenditure during running (Iaia et al. 2009); however, these results may differ during CC. Therefore, HIIET could be beneficial during CC, but priority should be given to RET when aiming to increase muscle mass with lower energy expenditure.

Regarding RET types, during eccentric RET the same muscle work can be produced with only ~ 15% of the metabolic demand, while cardiovascular demand is ~ 40% less when compared to concentric RET (Lastayo et al. 1999). These characteristics are attractive for implementation during CC, as eccentric RET can stimulate muscle hypertrophy but with much lower energy expenditure.

### Insulin resistance, dyslipidemia, and gut dysbiosis

Peripheral insulin resistance (Puppa et al. 2014) and gut bacteria dysbiosis (Bindels et al. 2016) are common CC mechanisms. Specifically, myofiber glucose uptake is insulin-dependent via GLUT4, but CC decreases muscle glucose uptake and GLUT4 expression, resulting in peripheral insulin resistance (Puppa et al. 2014). Additionally, metabolic dysregulation during CC involves lipid metabolism abnormalities, as indicated by abnormal blood lipid profiles (dyslipidemia) (Puppa et al. 2014).

A single 30-min low-frequency concentric-only electrical muscle stimulation session increased muscle GLUT4 mRNA levels (4.7-fold) in mice with CC, despite being suppressed by CC at rest (Puppa et al. 2014). Moreover, 6 weeks of concentric RET (loaded ladder climbing) increased GLUT4 expression in diabetic mice (Dehghan et al. 2016). Several studies demonstrated that LICET also increases GLUT4 expression in both skeletal and cardiac muscle (Bowman et al. 2021). Additionally, 6 weeks (3 week^−1^) of 4–6 sets of 30-s maximal bicycling increased muscle GLUT4 content by ~ 20% compared to baseline in healthy men (Burgomaster et al. 2007). Despite a lack of information regarding the effects of long-term exercise on muscle GLUT4 levels during CC, it appears that exercise modes that rely heavily on muscle glucose metabolism, such as concentric RET or endurance training, might be more effective than eccentric RET in increasing GLUT4 expression.

While the effects of exercise on insulin resistance during CC are unexamined, even a once-per-week (8 weeks), 30-min bout of eccentric RET (knee extensions) improved resting blood lipid profile (TG − 12.8%, TC − 8.8%, HDL-C 9.3%, LDL-C − 16.4%) and insulin sensitivity (resting glucose − 12%, insulin − 12%, homeostasis model assessment (HOMA) − 24%, HbA1C − 10.6%) in women, whereas equivalent concentric RET did not (Paschalis et al. 2011). Similar improvements were observed with elderly men after 12 weeks of once-per-week eccentric knee extension RET (resting glucose − 5%, insulin − 24%, HOMA − 28%, HbA1C − 6%, oral glucose tolerance test − 12%, TG − 16%, TC − 8%, HDL-C 12%, LDL-C − 7%), and elderly women after 12 weeks (2 week^−1^) of downstairs walking (TG − 20%, TC − 10%, HDL-C 10%, LDL-C − 13%, resting glucose − 9%, insulin − 18%, HOMA − 26%, HbA1C − 5%, oral glucose tolerance test − 11%), but these findings were not observed after equivalent concentric exercise (Chen et al. 2017a, b). These results are promising, and the effects might be further potentiated with greater weekly frequency and whole-body programs, however, this needs to be examined during CC.

Although dyslipidemia and insulin resistance are commonly counteracted through nutrition strategies by reducing body fat, exercise might be a preferable method, especially during CC. For example, after 12 weeks (3 week^−1^) of HIIET in men aiming to improve body composition, there was a 32% reduction in HOMA, and plasma insulin concentration (− 28%), without reductions in muscle mass (Matinhomaee et al. 2014). However, the group that underwent only dietary intervention without exercise had decreases in HOMA (10%) and plasma insulin concentration (12%), their muscle mass was also significantly decreased (2.2 kg) (Matinhomaee et al. 2014). Both groups reduced fat mass as the primary study aim, but it is important to note that CC patients are already encouraged to consume energy-dense foods (Arends et al. 2021), which may further dysregulate their blood lipid profile; thus, attempting to improve blood lipid profile and insulin resistance through nutrition alone can be suboptimal due to reductions in energy intake and muscle loss (Matinhomaee et al. 2014). These studies indicate that nutritional interventions alone are insufficient and might result in further muscle loss, whereas exercise can improve CC-induced metabolic dysregulation and muscle mass.

Gut dysbiosis has been traditionally addressed with nutritional and pharmacological interventions (Bindels et al. 2016), however, regular endurance exercise can confer benefits to gut microbiota. For example, 6 weeks (3 week^−1^) of LICET in sedentary women improved gut microbiota composition and function (Munukka et al. 2018). In another study, 6 weeks (3 week^−1^) of LICET in sedentary women improved gut microbiota composition and function regardless of diet, but those benefits were lost once exercise ceased (Allen et al. 2018). There is a lack of studies investigating the effects of RET on gut health, or comparing the effectiveness of LICET and HIIET, especially during CC, however, exercise seems promising for improving gut dysbiosis.

### Hypogonadism

Hypogonadism is the impaired gonad function (testes or ovaries), resulting in decreased sex hormone production, a symptom typically observed in both men and women with CC, with a prevalence of 70% in men with CC (Burney et al. 2012). Hypogonadism during cancer results in a deterioration in muscle mass, quality of life, and survival (Burney et al. 2012). In fact, blood testosterone levels in both men and women with CC inversely associate with overall survival (Bilir et al. 2015). A common method to treat low testosterone levels is testosterone treatment, which improves muscle mass, quality of life, and physical activity in both men and women with CC (Wright et al. 2018). However, a study in elderly hypogonadal men showed that exercise training was more effective than treatment in increasing aerobic capacity, and equally effective for muscle growth, leading the authors to conclude that exercise should be evaluated as an anti-aging intervention in preference to therapy (Chasland et al. 2021). A possible explanation is that testosterone receptor content in muscle is more important than serum testosterone levels for loading-induced muscle growth (Morton et al. 2018). Overall, there seems to be some evidence to suggest that testosterone administration alone for hypogonadism and muscle growth may be suboptimal, as low muscle testosterone receptor content may reduce treatment effectiveness. Moreover, there could be a role for exercise, when used synergistically with exogenous testosterone.

Despite multiple studies showing the effectiveness of both eccentric and concentric RET in increasing circulating free testosterone levels shortly after exercise (Kraemer et al. 2006), it is more clinically meaningful to examine resting level changes following long-term training, as these changes will exert chronic benefits. In a study with elderly hypogonadal men, 12 weeks of RET (3 week^−1^) and LICET increased resting blood testosterone levels (7%), but this improvement was not statistically significant, whereas the group that received testosterone therapy had a ~ 25% increase compared to the placebo group, irrespective of exercising or not (Chasland et al. 2021). Nevertheless, greater muscle loading increased muscle testosterone receptor content in animals, which in turn could amplify the signaling potential of circulating testosterone (Lee et al. 2003). This might explain the similar muscle growth (+ 0.7 kg) observed in both the exercise-only and the testosterone-only groups (despite the latter having higher serum testosterone levels), and the greater muscle growth in the testosterone-plus-exercise group (1.4 kg) versus the testosterone-only group (0.7 kg) (Chasland et al. 2021). These findings suggest that exercise training can not only increase serum testosterone but also enhance testosterone signaling potential for muscle growth, possibly through increasing muscle testosterone receptor content. However, the effectiveness of exercise training in improving hypogonadal status during CC remains to be examined.

### Impaired oxidative capacity and mitochondrial function

The muscle mitochondrial oxidative pathway, which is critical for cellular metabolism and growth, is profoundly impaired during CC by tumor-induced inflammation, leading to fatigue, weakness, and muscle protein breakdown (VanderVeen et al. 2017). Activation of transcription factors, such as NF-κB and STAT3, in muscle during CC leads to mitochondrial dysfunction (VanderVeen et al. 2017), and are relevant as therapeutic targets.

The STAT3 signaling pathway is considered important for muscle wasting in CC and other conditions with elevated IL-6, while STAT3 inhibition prevents CC in preclinical cancer models (Bonetto et al. 2012). Long-term eccentric RET might reduce the activation of STAT3. In mice with CC, only 2 weeks (4 week^−1^) of eccentric RET significantly reduced muscle STAT3 activation (Hardee et al. 2020). However, if this occurs in the muscles of humans with CC, and if equivalent concentric RET is similarly effective, remains uninvestigated.

Regarding NF-κB, in the brain of mice with Parkinson’s disease, 5 weeks (5 week^−1^) of loaded ladder climbing (typical rodent concentric RET protocol) decreased NF-κB expression (Kim et al. 2021). Moreover, 8 weeks (5 week^−1^) of HIIET was more effective than LICET in reducing NF-κB levels in mouse renal tissue after injury (Leite et al. 2021). In humans, eccentric RET increased inflammation to a greater degree than equivalent concentric RET, however repeating the same session does not trigger the same inflammatory response. For example, although circulating NF-κB levels increased after eccentric exercise in elderly men, repeating the same session after 8 weeks of eccentric lower-limb RET did not produce any significant changes (Jiménez-Jiménez et al. 2008). Repeated exercise not only protects against NF-κB elevations but may actively decrease it. This was shown in elderly women, where 12 weeks (3 week^−1^) of whole-body light-load RET (elastic bands) significantly reduced serum NF-κB levels (6%) (Roh et al. 2020). Moreover, circulating TLR4 levels (which lead to NF-κB activation and by themselves suppress protein synthesis) were down-regulated after 6 weeks (3 week^−1^) of eccentric knee-extension RET in healthy women (Fernandez-Gonzalo et al. 2014), however, there is a lack of information regarding changes after concentric RET. While these results appear promising, more research is needed to clarify the effects of exercise training on muscle NF-κB and STAT3 levels, particularly during CC.

Apart from transcription factors, it is important to examine changes in the final physiological properties of interest, namely mitochondrial content and oxidative capacity. Although a single 30-min concentric RET session did not improve mitochondrial biogenesis in mice with severe CC (Puppa et al. 2014), eccentric RET counteracted the overall muscle oxidative capacity loss (succinate dehydrogenase enzyme activity) and improved the distribution of oxidative-rich myofibers (Sato et al. 2019). Moreover, 2 weeks (4 week^−1^) of eccentric RET improved muscle oxidative capacity, and mitochondrial content (increased cytochrome-c oxidase enzyme activity) in CC muscle (Hardee et al. 2016, 2020), but the effects of concentric RET remain unexamined. Interestingly, increased basal protein synthesis was positively correlated with increased mitochondrial content (Hardee et al. 2020). These findings highlight the importance of controlling oxidative capacity and mitochondrial content in CC muscle for restoring protein synthesis.

It is well known that endurance training can improve oxidative capacity. Interestingly, only 14 days (6 total sessions) of 4–6 sets of 30-s maximal bicycling induced similar improvements to a group that was performing 90–120 min continuous bicycling in muscle oxidative capacity, buffering capacity (HIIET 7.6% vs LICET 4.2%), and glycogen content (HIIET 28% vs LICET 17%) (Gibala et al. 2006). Longer training periods produce similar results, as 6 weeks (3 week^−1^) of 4–6 sets of 30-s maximal bicycling induced similar improvements to a group that was performing 90–120 min continuous bicycling in mitochondrial biogenesis (PGC-1α levels), glycogen content, and oxidative capacity (Burgomaster et al. 2007, 2008). HIIET can produce similar muscle oxidative capacity and cardiovascular adaptations to LICET but in a more time- and energy-efficient manner, and thus may be preferable for improving muscle oxidative capacity and mitochondrial function.

Together, these studies indicate that RET and HIIET could be tested as an energy-efficient strategy to improve oxidative capacity and mitochondrial content during CC, although more research is required to examine this hypothesis.

### Side effects of cancer treatments

Exercise can not only counteract the health effects of CC but also the side effects of treatments. CC patients are often at advanced stages of their disease and will undergo multiple treatments to control cancer progression, but such treatments can cause serious side effects. As outlined below, several studies have established the safety and effectiveness of exercise in ameliorating treatment side effects.

Curative cancer treatment often involves surgery, which can cause further health complications and increase risk for other conditions. For example, after cancer-related surgeries for lung, prostate, and colorectal cancer, patients have increased risks of cardiovascular-related events, post-operative complications, and suffer large reductions in muscle mass and strength (Singh et al. 2017; Ashton et al. 2021). This can be particularly detrimental during CC, as it further accelerates muscle wasting, and significantly decreases survival (Choi et al. 2018). Exercise is safe and effective for improving aerobic exercise capacity, muscle strength, and health-related quality of life after prostatectomy (Singh et al. 2017; Ashton et al. 2021). For example, 6 weeks (2 week^−1^) of RET and 20 min of LICET increased muscle strength (24%), which was maintained post-surgery (Singh et al. 2017), however, it is impossible to make inferences about its effectiveness in attenuating the 2.7 kg loss of muscle experienced post-surgery, as there was no non-exercising group. In a similar study with the inclusion of a non-exercising group, exercise not only improved endurance exercise capacity and muscle strength after prostatectomy but also prevented increases in weight and fat mass occurring in the non-exercising group (Ashton et al. 2021). Systematic reviews have indicated that pre-surgical exercise for patients with prostate, lung, colorectal, colon, pancreatic, and bladder cancer may improve pre- and post-operative physical capacity, muscular strength, and quality of life, and decrease length of hospital stay, and post-operative complications (Singh et al. 2017).

Another common cancer-related treatment is chemotherapy which is a particularly critical topic during CC, as not only does muscle wasting during CC result in greater chemotherapy toxicity and dose reduction, but chemotherapy can cause CC or exacerbate existing CC (Brierley et al. 2019). Additionally, chemotherapy results in side effects, such as low energy, stress, nausea, and pain. Exercise can ameliorate these negative side effects (Johnsson et al. 2019). Notably, in women with breast cancer, 26 endurance exercise (both high- and moderate-intensities) and 31 RET sessions after chemotherapy onset improved energy and reduced nausea (Johnsson et al. 2019).

Radiation and hormone therapies are also common and often combined. Androgen-deprivation therapy decreases muscle and bone mass, physical function, and causes fatigue and metabolic syndrome (Galvão et al. 2008; Grossmann et al. 2011). These side effects are somewhat similar to the effects of CC, and can collectively increase the risk of falling, osteoporosis, and bone fractures and compromise quality of life (Galvão et al. 2008; Grossmann et al. 2011). Moreover, radiotherapy is commonly performed during androgen-deprivation therapy and can amplify the negative side effects of the latter, as radiotherapy can cause muscle atrophy, fibrosis, and fatigue (Kim et al. 2015). A combination of RET and LICET, performed either 2 or 3 times per week for 12 weeks prevented muscle loss in prostate cancer patients undergoing androgen-deprivation therapy, even when combined with radiotherapy (Newton et al. 2021).

Therefore, apart from benefits in health parameters relevant to CC, another reason to consider incorporating exercise during CC management is reduced treatment side effects.

## Application and future research directions

MEDLINE and Scopus databases were searched with published studies included until August 2021. Search terms included various combinations of: cachexia; sarcopenia; malnutrition; atrophy; muscle wasting; cancer; tumor; muscle; lean mass; exercise; training; physical activity. Secondary searches involved reference lists of eligible articles as well as systematic reviews and meta-analyses assessing interventions given to patients on ADT. The key criterion was to identify studies that included patients with substantial weight loss (≥ 5%) (Fearon et al. 2011) at the time of intervention, utilizing an exercise or multi-modal intervention, while including a measure of body mass or composition. From the identified studies (Table 1), we conclude that whole-body RET is safe and effective during CC, however, it is difficult to draw clear conclusions, as in half of those studies only some of the participants had CC, and many studies implemented a multi-modal program of exercise and nutrition. Nevertheless, a study implemented 6-month (2 week^−1^) whole-body RET either at home or performed under supervision in an exercise clinic, and half of the participants had pancreatic CC (Wiskemann et al. 2019). Even though there was higher adherence with home-based (78.4%) versus clinic-based (64.1%), only the clinic-based group significantly increased upper- and lower-body strength, and body mass (3.1%). Notably, the RET was of typical intensity (50–80% of the participant’s maximum strength) and volume (3 sets; 8–12 repetitions) to induce muscle hypertrophy in healthy populations (Wiskemann et al. 2019). In another study, patients with pancreatic CC underwent 12 weeks (2 week^−1^) of whole-body RET, similar to the previous study (50–80% of the participant’s 1-repetition maximum; 3 sets; 8–12 repetitions) (Kamel et al. 2020). There were improvements in mobility, muscle mass (1–2%), and strength, of both upper- and lower-limbs compared to a non-exercising group. Therefore, even higher-intensity (80% intensity) whole-body RET typically prescribed for healthy populations is safe and effective during CC and could be safely implemented in future research.

**Fig. 2:**
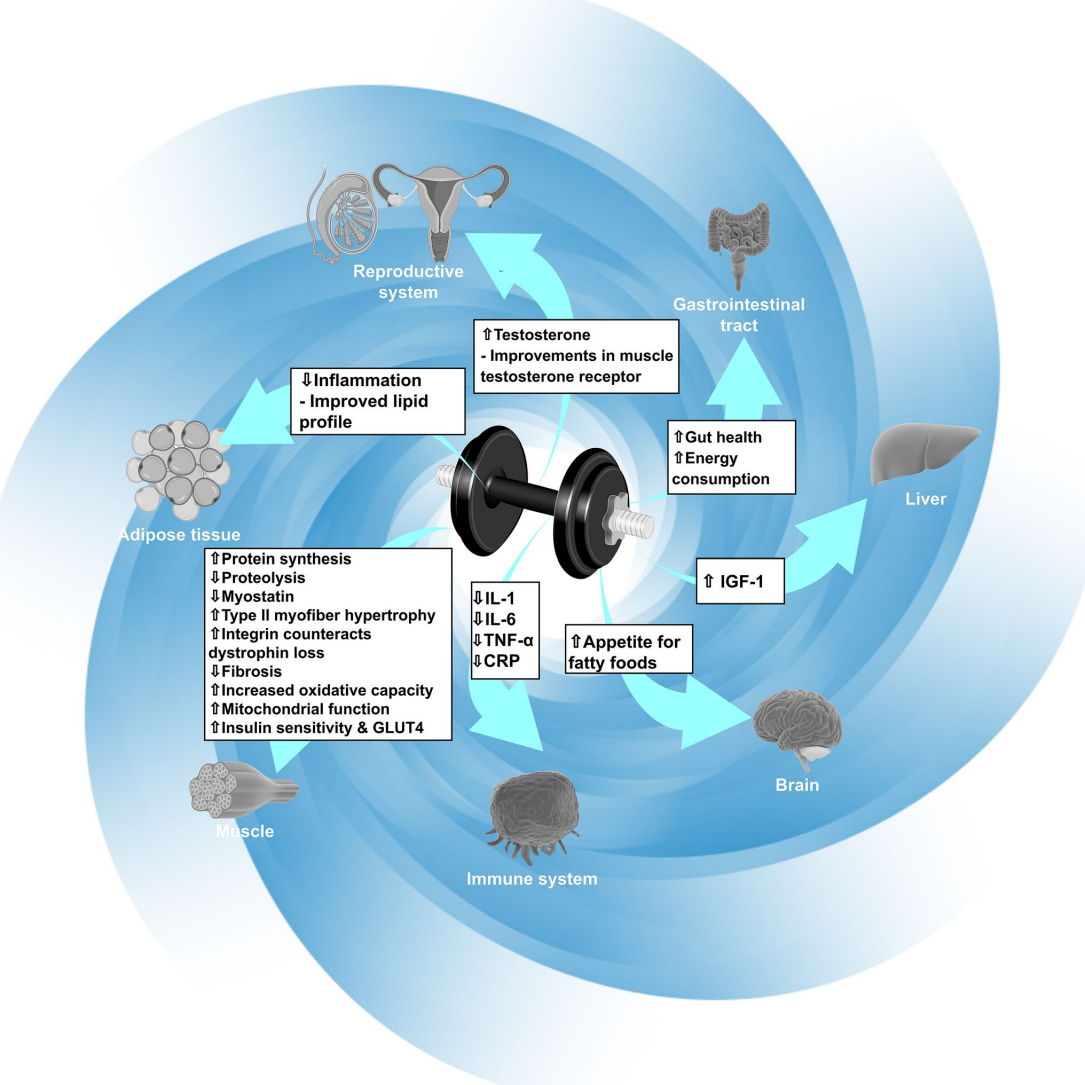
Reversal of the cachexia feedback loop by targeted resistance-based exercise training. *CRP* C-reactive protein, *IGF-1* insulin growth factor 1, *IL* interleukin, *TGF-β* transforming growth factor beta, *TNF-α* tumor necrosis factor alpha

The information included in this review suggests that RET (see Fig. 2), and specifically types focusing on the eccentric portion of the movement, are potentially more tolerable, efficient, and effective in counteracting CC mechanisms, and increasing muscle mass when compared to endurance training. Apart from greater muscle growth at lower energy costs, eccentric RET is also more time-efficient, as only 20–30 min of eccentric RET per week can elicit large increases (> 30%) in muscle size (Mavropalias et al. 2021b). However, researchers should be aware that unaccustomed high-intensity eccentric RET can induce muscle soreness, decreased muscle strength, and increased circulating levels of intramuscular proteins such as creatine kinase (Mavropalias et al. 2021a). Consequently, we recommend that intensity, in either concentric or eccentric RET, is increased over several sessions, while submaximal exercise (pre-conditioning) can protect against future higher-intensity exercise (Mavropalias et al. 2020). As eccentric RET appears to be more specific and potent for counteracting the multiple causes of wasting at the myofiber level than conventional or concentric RET, could be very beneficial if incorporated as an additional brief component after a conventional RET program.

Moreover, there is unexplored potential of repeated RET (particularly eccentric) in ‘exploiting’ elevated inflammation to proportionately increase muscle growth. As yet, the effectiveness of exercise training in improving inflammation, as well as appetite, insulin resistance, dyslipidemia, and hypogonadism, in patients with CC remains unexamined. Further, a combination of RET and HIIET might improve hunger, total energy consumption, oxidative capacity, mitochondrial content, and gut microbiota composition and function. Well-controlled trials involving patients with CC are required to answer these research questions.

It is preferable that research projects are conducted in a clinical setting with appropriate exercise equipment, as it is otherwise difficult to elicit clinically meaningful muscle growth with body weight exercises alone, especially when unsupervised. This was shown in a study where only the CC patients who underwent supervised whole-body RET increased upper- and lower-body strength, and body mass (Wiskemann et al. 2019). While CC patients have lower muscle strength and exercise capacity compared to healthy individuals, the exercise principles for muscle growth must be still adhered to; it is well established that sufficient intensity and volume, and progressive manipulation of these parameters are required for muscle growth (Grgic et al. 2019), which may be challenging with light-load or bodyweight-only exercises. Moreover, adequate supervision cannot be provided to the same extent during home-based exercise.

Choosing suboptimal exercise modes can be counterproductive, and could further contribute to CC without impacting muscle growth. For example, LICET can increase energy expenditure, fat loss, and mitochondrial biogenesis in adipose tissue (Grgic et al. 2019; McKie and Wright 2020), and although these would be desirable in healthy individuals, these are CC mechanisms and could exacerbate the disease, and thus progressive RET should be focused. If progressive HIIET is incorporated in research, the intensity should be closer to the higher spectrum of the participant’s individual exercise capacity (e.g., assessed through perceived effort), and not ‘high intensity’ as defined based on healthy persons. Moreover, HIIET should be performed on exercise equipment in which the patient is safely secured, without risking falls or being unable to stop immediately, such as recumbent bikes for the lower limbs, and sitting arm cranks or rowing ergometers for the upper limbs. The patient’s perceived effort, heart rate, and signs of faintness or pain should be closely monitored by exercise professionals.

## Conclusion

Cachexia is a common and devastating symptom of many chronic conditions. In this review, the multiple disease mechanisms and signs were outlined and these are often complementary and amplify one another. Additionally, evidence from the available literature indicates that exercise therapy could potentially counteract most of the CC mechanisms and side effects from cancer-related treatments commonly administered during CC. Prevention and early treatment of CC have been extensively recommended (Fearon et al. 2011), but in many cases, prevention is not possible, as the patient is already in mid-CC when diagnosed (Fearon et al. 2011), highlighting the need for more trials in advanced CC stages. To date, most of the available evidence is limited to animal models of CC or healthy humans, although this still provides valuable information. Nevertheless, the available evidence is still limited, while exercise during CC has been discouraged (Roeland et al. 2020), despite its success in increasing muscle and body mass in humans with particularly aggressive CC forms (Lønbro et al. 2013a; Naito et al. 2019; Wiskemann et al. 2019; Kamel et al. 2020). As a result, there is an urgent need for human trials to examine the effectiveness of RET in counteracting CC mechanisms and increasing muscle mass.

As outlined in the sections above, due to the increased energy expenditure, adipose tissue mitochondrial biogenesis, and muscle wasting typically observed during CC, LICET is discouraged, and instead different forms of RET should be investigated to determine their effectiveness in counteracting muscle wasting but adjusted to the needs and capacity of the patient. Regarding specific RET types, eccentric RET appears promising in increasing protein synthesis regardless of elevated inflammation, resulting in muscle growth at lower energy costs. However, even conventional eccentric/concentric RET, when progressively increased in volume and intensity, is a potent, easily accessible, low-cost, and safe therapy for research in the management of CC-induced muscle wasting.

## Abbreviations

CC: Cancer cachexia
CRP: C-reactive protein
HIIET: Higher-intensity interval endurance training
HOMA: Homeostasis model assessment
IGF-1: Insulin-like growth factor 1
LICET: Low-intensity continuous endurance training
mTORC1: MTOR complex 1
RET: Resistance exercise training
TNF-α: Tumor-necrosis factor alpha
